# Patterns of Bone Failure in Localized Prostate Cancer Previously Irradiated: The Preventive Role of External Radiotherapy on Pelvic Bone Metastases

**DOI:** 10.3389/fonc.2019.00070

**Published:** 2019-02-15

**Authors:** Mathieu Grapin, Magali Quivrin, Aurélie Bertaut, Etienne Martin, Luc Cormier, Sylvain Ladoire, Alexandre Cochet, Gilles Créhange

**Affiliations:** ^1^Department of Radiation Oncology, Unicancer-Georges-Francois Leclerc Cancer Center, Dijon, France; ^2^Methodology, Data-Management, and Biostatistics Unit, Unicancer-Georges-Francois Leclerc Cancer Center, Dijon, France; ^3^Department of Urology, University Hospital of Dijon, Dijon, France; ^4^Department of Medical Oncology, Unicancer-Georges-Francois Leclerc Cancer Center, Dijon, France; ^5^Department of Nuclear Medicine, Unicancer-Georges-Francois Leclerc Cancer Center, Dijon, France; ^6^Le2i FRE2005, Arts et Métiers, Université Bourgogne Franche-Comté, Dijon, France

**Keywords:** prostate cancer, radiotherapy, surgery, metastasis, patterns of failure

## Abstract

**Introduction:** External beam radiation therapy (EBRT) can cure localized prostate cancer (PCa) by sterilizing cancer cells in the prostate gland and surrounding tissues at risk of microscopic dissemination. We hypothesized that pelvic EBRT for localized PCa might have an unexpected prophylactic impact on the occurrence of pelvic bone metastases.

**Material and Methods:** We reviewed the data of 332 metastatic PCa patients. We examined associations between the number (≤5 vs. >5) and the location of bone metastases (in-field vs. out-of-field), which occurred at first relapse, and a previous history of EBRT for PCa (EBRT vs. No-EBRT).

**Results:** One hundred and ten patients M0 at baseline were eligible. Fifty-six patients (51%) were in the No-EBRT group, and 54 patients (49%) in the EBRT group. The proportion of patients who developed >5 bone metastases in the bony pelvis was higher in the No-EBRT group vs. the EBRT group: 10 patients (18%) vs. 2 patients (4%), respectively (*p* = 0.02). By multivariate analysis EBRT was associated with a lesser occurrence of patients who had >5 bone metastases in the bony pelvis (OR = 0.17 [95%CI, 0.04–0.87], *p* = 0.03). Time to occurrence of bone metastases ≥5 years (OR = 0.10 [95%CI, 0.05–0.19], *p* < 0.01), prior curative prostate treatment (OR = 0.58 [95%CI, 0.36–0.91], *p* = 0.02), >5 bone metastases in bony pelvis (OR = 2.61 [95%CI, 1.28–5.31], *p* < 0.01), >5 bone metastases out of bony pelvis (OR = 1.73 [95%CI, 1.09–2.76], *p* = 0.02) were all predictive of overall survival.

**Conclusion:** Previous pelvic EBRT for PCa is associated with a lower number of pelvic bone metastases, which is associated with better overall survival.

## Introduction

Prostate irradiation with External Beam Radiotherapy (EBRT) is a standard treatment for localized and locally advanced prostate cancer (PCa) (1, 2). Androgen deprivation therapy (ADT) concomitantly with EBRT has become the standard for intermediate, high-risk, or locally advanced PCa, improving local control, disease free survival (DFS), overall survival (OS), and decreasing distant metastases (3, 4).

For locally advanced PCas, EBRT improves DFS, cancer specific survival and OS when associated with ADT compared to ADT alone (5, 6). Mottet et al. suggested that EBRT combined with ADT in this setting was able to reduce distant failures compared to ADT alone (7).

This benefit of ADT and EBRT on distant metastases could be explained by a prophylactic effect of each therapeutic modality on micro metastases, or enhanced local control possibly resulting in a diminished wave of metastases in case of local relapse.

The radiation therapy oncology group (RTOG) 94-13 phase III trial showed that whole pelvic nodal radiation therapy (WPRT) improved progression-free survival (PFS) (8). Nevertheless, this was not confirmed by final analysis (9, 10).

In the postoperative setting, EBRT of the prostatic fossa alone (i.e., without WPRT or ADT) was shown to provide better biochemical and clinical DFS rates (11) up to 10 years and beyond (12), with significantly improved metastasis-free survival (MFS) in the south west oncology group (SWOG) trial (13).

The bony pelvis is a favored site for PCa bone metastases (14, 15). Additionally, patients who develop pelvic bone metastases may have worse OS (16).

It remains debated whether there is an additional benefit of pelvic EBRT on hypothetical nodal micro-metastases in patients with high-risk localized PCa. We hypothesized that pelvic EBRT for localized PCa may have an unexpected prophylactic impact on the occurrence of pelvic bone metastatic burden (in-field metastases) compared to patients who did not undergo pelvic EBRT.

## Materials and Methods

We reviewed the data of 332 patients who were referred between January 1986 and December 2012 to our cancer center for metastatic PCa and who had a bone scan with bone metastases from their PCa. Patients were included in EBRT group if they had received EBRT of the prostate or postoperatively after radical prostatectomy (RP) with or without WPRT. Thus, in EBRT group we differentiated prostate or prostate bed radiotherapy (PPBR) ± WPRT. Patients were defined as “N1” if they had clinical lymph node involvement or pathological lymph node involvement.

We recorded the number of bone metastases observed on bone scan performed at the time of bone metastatic relapse, notably whether bone metastases were in bony pelvis or not. The bone metastases were counted inside and outside the bony pelvis (1–5 bone metastases, of which each was numbered and reported; or >5 bone metastases). Patients with a super bone scan had a very high number of diffuse and/or contiguous bone metastases so that it cannot be numbered (i.e., >5). A definition based on up to 5 detectable lesions is widely employed and was used in our study to define an oligometastatic disease (17, 18).

**Figure 1:**
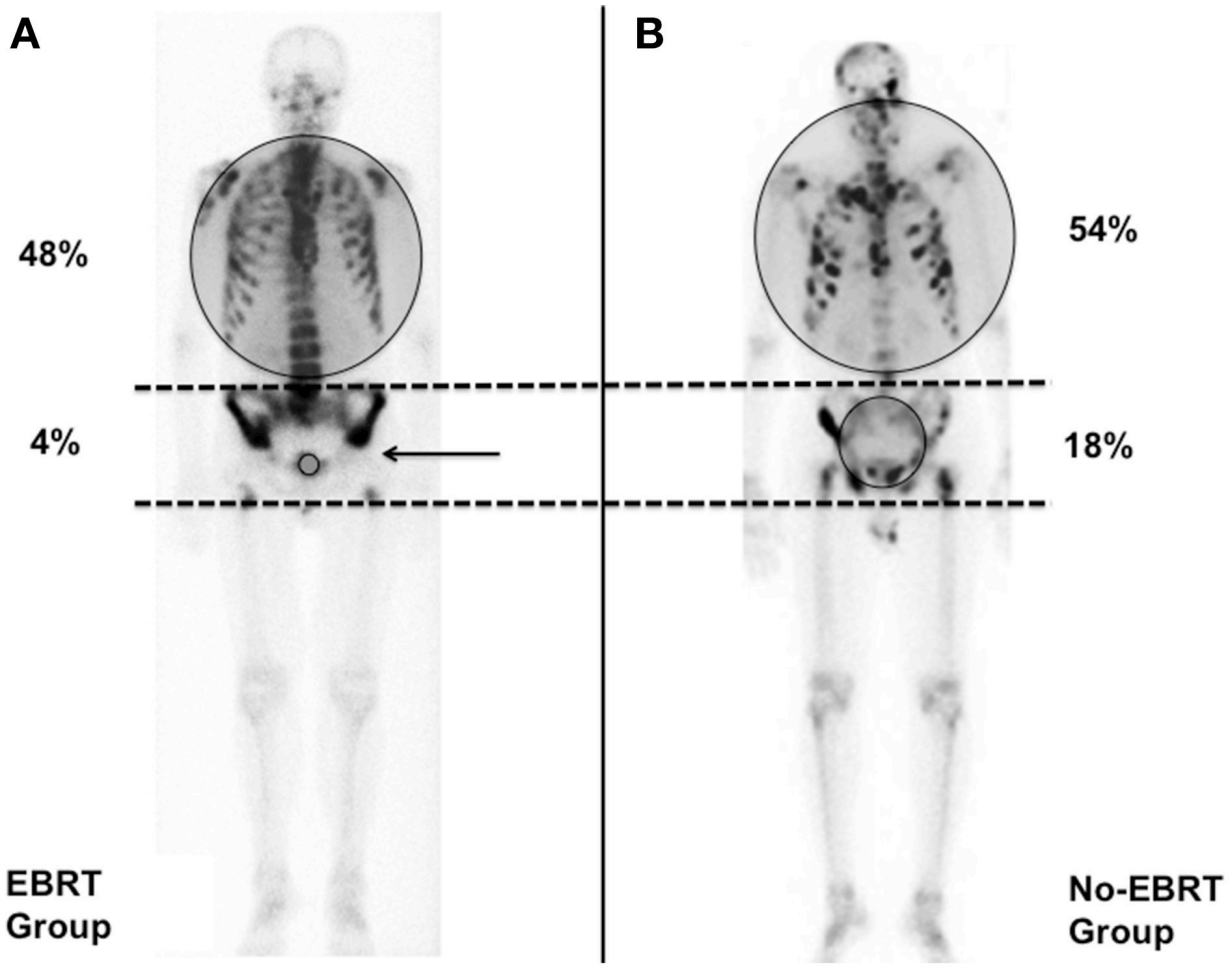
Pattern of occurence of bone metastases >5 in the external beam radiation therapy (EBRT) and No-EBRT-groups. Gray circles indicate the proportion of bone metastases > 5 inside and outside the pelvis. **(A)** Bone scan from a patient in the EBRT group who developed multimetastatic disease outside the pelvis. The pelvis is defined as the bone anatomy between the 2 dotted lines. The black arrow shows the limit of the prostate or prostate bed radiation (PPBR) field. There is a clear decrease of the bone uptake in-field. **(B)** Bone scan from a patient in the No-EBRT group. This man developed a multimetastatic disease including the pelvis.

Bone metastases in pelvic nodal radiation fields were defined as any bone metastasis found between L5-S1 and the upper border of pubic symphysis. Bone metastases in PPBR fields were defined as any bone metastasis that occurred between the upper border of pubic symphysis and the lesser trochanter of the femoral heads as shown in Figure 1. Bone metastases of the bony pelvis included bone metastases of the pelvic nodal field and PPBR field. The number (1 to 5 or >5 bone metastases) and location (in-field vs. out-of-field) of each bone metastasis were analyzed according to whether or not the patient previously received treatment (PPBR ± WPRT vs. no EBRT for PCa).

### Statistical Analysis

Quantitative variables are described as mean ± standard deviation (SD) in case of normal distribution or median (range) in case of non-normal distribution. Qualitative variables are described as number (percentage) after exclusion of missing data. Comparisons between EBRT and No-EBRT groups were performed using the Student *T*-test, Wilcoxon test, Chi square or Fisher's exact test, as appropriate. Median follow up was determined using the reverse Kaplan Meier method. Overall survival rates and median survival were calculated from initial diagnosis using the Kaplan Meier method and compared between groups using the log rank test. Variables associated with the occurrence of more than 5 metastases in the bony pelvis were determined by multivariate logistic regression. Predictors of OS were determined using univariate and multivariate Cox proportional hazards models. Odds ratios (OR) and hazard ratios (HR) are given with the associated 95% confidence interval (95%CI). The same statistical analyses were used to identify factors associated with the occurrence of more than 1 metastasis in the bony pelvis. For multivariate models, all predictors with a *p*-value <0.20 by univariate analysis were included in the multivariate model. The correlation between the variables was tested using the Pearson correlation coefficient. The final models include predictors with a *p*-value <0.05. Correlations between variables eligible for multivariate models were tested. Test were 2-sided and *p*-value <0.05 was considered statistically significant. All analyses were performed using SAS version 9.4 (SAS Institute Inc., Cary, NC, USA).

## Results

### Description of Study Population and Treatments

Over the study period, 332 patients were referred to our cancer center for metastatic PCa. Two hundred and twenty two patients were excluded for the following reasons: bone metastases (M1) at initial diagnosis (115 patients, 52%); second cancer (63 patients, 28%); prostate brachytherapy (3 patients, 1%); visceral metastases (3 patients, 1%), or missing data (38 patients, 17%). In total, 110/332 patients (33%) with localized PCa at initial diagnosis, and initially treated with curative intent were included: 56 patients (51%) did not undergo any EBRT (No-EBRT group) and 54 patients (49%) received PPBR ± WPRT (EBRT group).

As shown in Table 1, the risk group at baseline, Gleason score and lymph node staging were not different between the No-EBRT and EBRT groups. Median prostate specific antigen (PSA) at initial diagnosis was significantly higher in the No-EBRT group (23.0 ng/ml [4.5–297.7] vs. 10.4 ng/ml [1.2–290.0] in the EBRT group, *p* < 0.01). Median age at initial diagnosis was 70 years [51–86] in the No-EBRT group and 67 years [49–80] in the EBRT group (*p* < 0.01).

**Table 1:** Baseline characteristics of the study population.

	**No-EBRT (*N* = 56) *N* (%)**	**EBRT (*N* = 54) *N* (%)**	***p*-value**
**Age**			
Median [min–max]	70 [51–86]	67 [49–80]	< 0.01***
**INITIAL DISEASE STAGE**			
**Node involvement**			
N0 - Nx	37 (93)	44 (92)	0.89*
N1	3 (7)	4 (8)	
Missing	16	6	
**Risk group**			
Low	2 (5)	2 (4)	1.0*
Intermediate	11 (25)	12 (24)	
High	31 (71)	36 (72)	
Missing	12	4	
**Gleason score**			
≤6	16 (38)	16 (32)	0.30**
7	12 (29)	22 (44)	
≥ 8	14 (33)	12 (24)	
Missing	14	4	
**PSA**			
Median [min–max]	23.0 [4.5–297.7]	10.4 [1.2–290.0]	<0.01***
**TREATMENT AT INITIAL DIAGNOSIS**			
RP alone	9 (16)	–	
ADT alone	39 (70)	–	
RP + ADT	8 (14)	–	
EBRT alone	–	9 (17)	
RP + EBRT	–	3 (6)	
ADT + EBRT	–	29 (54)	
RP + ADT + EBRT	–	13 (24)	
PLND	10 (18)	21 (39)	0.01**

* *Fisher test*,

** Chi-2 test

*** *Wilcoxon tests*.

*ADT, Androgen Deprivation Therapy; EBRT, External Beam Radiation Therapy; MV, Missing Values; PLND, Pelvic Lymph Node Dissection; PSA, Prostate-Specific Antigen; RP, Radical Prostatectomy*.

*p-values < 0.05 are considered statistically significant, and are in bold*.

In the EBRT group, 29 patients (54%) had ADT with EBRT, 13 patients (24%) received EBRT and ADT after RP, 9 patients (17%) had EBRT alone, and 3 patients (6%) received EBRT after RP. In the No-EBRT group, 39 patients (70%) had ADT alone, 9 patients (16%) were treated by RP alone, and 8 patients (14%) received ADT after RP (Table 1). The rate of Pelvic Lymph Node Dissection (PLND) was higher in the EBRT group [21 (39%) vs. 10 patients (18%) in the No-EBRT group, *p* = 0.01].

In the EBRT group, 24 patients (44%) received PPBR + WPRT and 30 patients (56%) received PPBR. The median total dose of WPRT was 46 Gy [40–60], while the median total dose of PPBR was 70 Gy [60–80]. All the patients who received WPRT also received PPBR. Fifty-one patients (94%) were irradiated with static fields such as 3D-EBRT or intensity modulated radiation therapy (IMRT), whereas only 3 patients (6%) were irradiated with volumetric modulated arc therapy (VMAT).

### Distribution of Bone Metastases With Respect to Radiation Fields

The median PSA value at the time of bone relapse was not significantly different between the No-EBRT and EBRT groups: 27 ng/ml [0.1–1356] and 21 ng/ml [0.3–4737], respectively (*p* = 0.22) (Table 2). The median time between initial diagnosis and occurrence of bone metastases was not significantly different between the No-EBRT and EBRT groups: 3.7 years [0.6–18.0] vs. 5.1 years [1.0–15.5], respectively (*p* = 0.11). Patients in the EBRT group were more likely to develop oligometastases (≤5) at relapse (52%), while patients in the No-EBRT group at baseline had a higher number of metastases at relapse (>5) (63%), although the difference was not significant (*p* = 0.13). There was no significant difference between groups in terms of the proportion of patients who developed 0 to 5 bone metastases or bone polymetastatic disease (>5) outside the bony pelvis (*p* = 0.57).

**Table 2:** Description of the incidence of bone metastases according to the treatment group.

	**No-EBRT group *N* = 56**	**EBRT group *N* = 54**	***p*-value**
**TIME BETWEEN DIAGNOSIS AND OCCURRENCE OF BONE**			
**METASTASES (YEARS)**			
Median [range]	3.7 [0.7–18.0]	5.1 [1.0–15.5]	0.11*
**PSA AT RELAPSE (ng/ml)**			
Median [range]	27.0 [0.1–1356.0]	21.0 [0.3–4737.0]	0.22*
**NUMBER OF BONE METASTASES**			
[1−5]	21 (38%)	28 (52%)	0.13**
>5	35 (63%)	26 (48%)	
**NUMBER OF BONE METASTASES OUTSIDE OF THE BONY PELVIS**			
[0−5]	26 (46%)	28 (52%)	0.57**
>5	30 (54%)	26 (48%)	
**NUMBER OF BONE METASTASES IN THE BONY PELVIS**			
[0−5]	46 (82%)	52 (96%)	0.02**
>5	10 (18%)	2 (4%)	
**NUMBER OF BONE METASTASES INSIDE THE PELVIC NODAL FIELD**			
[0−5]	51 (91%)	52 (96%)	0.44***
> 5	5 (9%)	2 (4%)	
**NUMBER OF BONE METASTASES INSIDE THE PPBR FIELD**			
[0−5]	53 (95%)	54 (100%)	0.24***
> 5	3 (5%)	0 (0%)	

* *Mann-Whitney test*,

** Chi-2 test

*** *Fisher test*.

*EBRT, External beam radiation therapy; PPBR, Prostate or prostate bed radiotherapy; PSA, Prostate-specific antigen*.

*p-values < 0.05 are considered statistically significant, and are in bold*.

The number of patients who developed >5 bone metastases in the bony pelvis was significantly higher in the No-EBRT group compared to the EBRT group: 10 patients (18%) vs. 2 patients (4%) respectively, (*p* = 0.02). No such difference was observed for the pelvic nodal field: 5 patients (9%) had >5 bone metastases vs. 2 patients (4%), respectively (*p* = 0.44); or for the PPBR field: 3 patients (5%) had >5 bone metastases vs. 0, respectively (*p* = 0.24).

Although there was no significant difference in the proportion of patients who developed >1 bone metastasis in the pelvic nodal field [24 patients (43%) in the No-EBRT group vs. 16 (30%) in the EBRT group, *p* = 0.15], the number of patients who developed >1 bone metastasis in the PPBR field was higher in the No-EBRT group than in the EBRT group: 19 (34%) vs. 1 patient (2%) respectively (*p* < 0.01).

As shown in Table 3, by multivariate analysis, the following variables were significantly associated with the occurrence of >5 bone metastases in the bony pelvis: EBRT (OR = 0.17 [95% CI, 0.04–0.87], *p* = 0.03), > 5 bone metastases outside of the bony pelvis (OR = 13.18 [95% CI: 1.61–108.24], *p* = 0.02).

**Table 3:** Univariate and multivariate analyses of the factors associated with the occurrence of >5 bone metastases in the bony pelvis.

		**Univariate analysis**				**Multivariate analysis**			
		**B-mets> 5/Total**	**OR**	**95% CI**	***p*-value**	**B-mets > 5/Total**	**OR**	**95% CI**	***p*-value**
**YEAR OF DIAGNOSIS**									
	[1987; 2005]	8/76	1						
	[2005; 2012]	3/33	0.85	[0.21–3.43]	0.82				
**TIME BETWEEN DIAGNOSIS AND OCCURRENCE OF B-METS (YEARS)**									
	≤ 5	6/59	1						
	> 5	5/50	0.98	[0.28–3.43]	0.98				
**LYMPH NODE STAGING**									
	N0	7/81	1						
	N1	0/7	0.66	[0.03–153.49]	0.80				
**AGE AT DIAGNOSIS (YEARS)**									
	< 70	5/63	1						
	≥ 70	6/46	1.74	[0.50–6.09]	0.39				
**RISK GROUP**									
	Low or intermediate	2/27	1						
	High	7/67	1.46	[0.28–7.51]	0.66				
**GLEASON SCORE**									
	≤ 6	2/32	1						
	> 6	4/60	1.07	[0.19–6.19]	0.94				
**INITIAL PEAK PSA BEFORE TREATMENT (ng/ml)**									
	< 15	3/45	1						
	≥ 15	4/44	1.40	[0.30–6.65]	0.67				
**EBRT**									
	No	10/56	1			10/56	1		
	Yes	2/54	0.18	[0.04–0.85]	**0.03**	2/54	0.17	[0.04–0.87]	**0.03**
**TYPE OF EBRT**									
	Static fields (3D-EBRT or IMRT)	2/51	1						
	VMAT	0/3	0.35	[0.01–13.67]	0.58				
**RP**									
	No	9/77	1						
	Yes	3/33	0.76	[0.19–2.99]	0.69				
**LYMPHADENECTOMY**									
	No	9/72	1						
	Yes	2/31	0.48	[0.10–2.38]	0.37				
**NUMBER OF B-METS OUTSIDE OF THE BONY PELVIS**									
	[0–5]	1/54	1			1/54	1		
	> 5	11/56	12.96	[1.61–104.26]	**0.02**	11/56	13.18	[1.61–108.24]	**0.02**
**CURATIVE TREATMENT (EBRT AND/OR RP)**									
	No	9/39	1						
	Yes	3/71	0.15	[0.04–0.58]	**<0.01**				

*Since the variables “External Beam Radiation Therapy (EBRT)” and “Curative treatment [EBRT and/or Radical Prostatectomy (RP)]" were correlated, only the variable “EBRT” was included in the multivariate model*.

*B-mets, Bone metastases; CI, Confidence Interval; EBRT, External Beam Radiation Therapy; IMRT, Intensity Modulated Radiation Therapy; OR, Odds Ratio; PSA, Prostate Specific Antigen; RP, Radical Prostatectomy; VMAT, Volumetric Modulated Arc Therapy*.

*p-values < 0.05 are considered statistically significant, and are in bold*.

### Overall Survival

**Figure 2:**
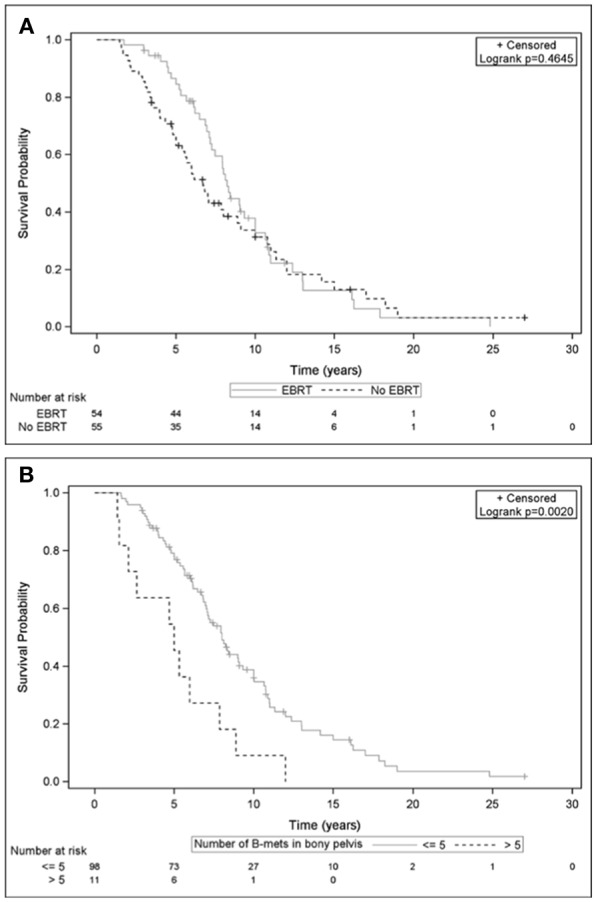
Overall survival curves from initial diagnosis, among patients with localized prostate cancer according to: **(A)** Whether they received External Beam Radiation Therapy (EBRT group) or not (No-EBRT group). **(B)** Presence of ≤5 bone metastases compared to >5 bone metastases in the bony pelvis at first bone relapse.

The median follow up was 7.9 years [6.8–8.9]. In the No-EBRT group, median OS was 6.7 years [5.0–8.9]; vs. 8.2 years [7.2–10.0] in the EBRT group (*p* = 0.46) (Figure 2A). As shown in Figure 2B, median OS was significantly higher when patients had ≤ 5 bone metastases in bony pelvis compared to >5 bone metastases in the bony pelvis, 8.0 [7.0–15.0] vs. 5.0 years [1.6–7.9], respectively (*p* < 0.01).

In multivariate analysis (Table 4), the following variables were significantly predictive of OS: curative treatment (EBRT and/or RP) OR = 0.58 [95% CI, 0.36–0.91] (*p* = 0.02), delay between diagnosis and occurrence of bone metastases ≥ 5 years (OR = 0.10 [95% CI: 0.05–0.19], *p* < 0.01), number of bone metastases in bony pelvis >5 (OR = 2.61 [95% CI: 1.28–5.31], *p* < 0.01), number of bone metastases out of bony pelvis > 5 (OR = 1.73 [95% CI: 1.09–2.76], *p* = 0.02).

**Table 4:** Univariate and multivariate analysis of the factors associated with overall survival (Cox model).

		**Univariate analysis**				**Multivariate analysis**			

		**Death/total**	**HR**	**95% CI**	***p*****-value**	**Death/Total**	**HR**	**95% CI**	***p*****-value**
**YEAR OF DIAGNOSIS**									
	[1986; 2005]	69/76	1						
	[2005; 2012]	19/33	2.27	[1.30–3.97]	**<0.01**				
**AGE AT DIAGNOSIS (YEARS OLD)**									
	< 70	47/63	1						
	≥70	41/46	1.91	[1.24–2.94]	**<0.01**				
**LYMPH NODE STAGING**									
	N0-Nx N1	63/81 6/7	1 0.65	[0.28–1.53]	0.32				
**GLEASON**									
	≤ 6	27/32	1						
	>6	46/60	1.37	[0.84–2.22]	0.20				
**PSA BEFORE TREATMENT (ng/ml)**									
	≤ 15	34/45	1						
	>15	39/44	1.06	[0.66–1.69]	0.82				
**D'AMICO SCORE**									
	Low or intermediate risk	19/27	1						
	High risk	57/67	1.76	[1.03–2.99]	**0.04**				
**EBRT**									
	No	45/55	1						
	Yes	43/54	0.85	[0.56–1.30]	0.46				
**RP**									
	No	67/76	1						
	Yes	21/33	0.52	[0.32–0.85]	**<0.01**				
**LYMPHADENECTOMY**									
	No	56/71	1						
	Yes	27/31	0.78	[0.49–1.26]	0.31				
**CURATIVE TREATMENT (EBRT AND/OR RP)**									
	No	34/38	1			34/38	1		
	Yes	54/71	0.48	[0.31–0.74]	**<0.01**	54/71	0.58	[0.36–0.91]	**0.02**
**DELAY BETWEEN DIAGNOSIS AND OCCURRENCE OF BONE METASTASES (YEARS)**									
	< 5	45/59	1			45/59	1		
	≥ 5	43/50	0.14	[0.08–0.25]	**<0.01**	43/50	0.10	[0.05–0.19]	**<0.01**
**NUMBER OF BONE METASTASES**									
	[0–5]	34/49	1						
	> 5	54/60	1.67	[1.08–2.58]	**0.02**				
**NUMBER OF BONE METASTASES IN BONY PELVIS**									
	[0–5]	77/98	1			77/98	1		
	> 5	11/11	2.64	[1.39–5.02]	**<0.01**	11/11	2.61	[1.28–5.31]	**<0.01**
**NUMBER OF BONE METASTASES IN PELVIC NODAL FIELD**									
	[0–5]	82/103	1						
	> 5	6/6	2.86	[1.23–6.65]	**0.02**				
**NUMBER OF BONE METASTASES IN PPBR FIELD**									
	[0–5]	86/107	1						
	> 5	2/2	2.21	[0.54–9.09]	0.27				
**NUMBER OF BONE METASTASES OUT OF BONY PELVIS**									
	[0–5]	39/54	1			39/54	1		
	> 5	49/55	1.40	[0.92–2.14]	0.12	49/55	1.73	[1.09–2.76]	**0.02**

*Variables significant at p < 0.20 in the univariate model were included in the multivariate model*.

*Correlation between variables was tested beforehand*:

*-The year of diagnosis and the time to occurrence of bone metastases were correlated; only the time to occurrence of bone metastases was retained for multivariate analysis*.

*-The variables “Radical Prostatectomy (RP)” and the “curative treatment [External Beam Radiation Therapy (EBRT) and/or RP] were also correlated; the variable “Curative treatment” was retained for analysis*.

*- For the numbers of bone metastases, only the total number of bone metastases in the bony pelvis and out of the bony pelvis was retained*.

*The threshold for retention in the final model was p < 0.05*.

*CI, Condifence Interval; HR, Hazard Ratio; PPBR, Prostate or Prostate Bed Radiotherapy; PSA, Prostate Specific Antigen*.

*p-values < 0.05 are considered statistically significant, and are in bold*.

## Discussion

We found that PPBR ± WPRT EBRT were associated with a lower number of bone metastases in the bony pelvis compared to other treatments. The association between local failure and distant metastases in PCa can be explained by two different hypotheses (19, 20). The first is the patho-biological aggressiveness theory, which states that some tumors are more virulent, and thus most difficult to eradicate locally and more inclined to have micrometastases at diagnosis. The second is the reseeding theory, which states that for localized tumors without micrometastases at diagnosis, the failure to completely eradicate the tumor may lead to subsequent shedding of tumor cells and a late wave of metastases.

To explain our findings, one possible explanation is that the bone receiving the radiation dose could be the site of micrometastatic disease at initial diagnosis, thus supporting the pathobiological aggressiveness theory. Indeed, when EBRT is used to treat local PCa, bone tissue, which gives the appearance of being free from metastasis, receives a range of unintended doses. Another hypothesis is that EBRT may induce fibrosis in bone tissue which can modify bone vascularization, making the irradiated bone impervious to bone metastases.

The origin of our study comes from a simple but surprising observation from nuclear medicine physicians, namely that in some patients who develop bone involvement many years after prostate and/or pelvic EBRT, the bony pelvis that was partially or totally within the radiation fields is often free of bone metastases, with a decrease in the physiological metabolic activity of the skeleton on the bone scan (Figure 1).

Our results showed that PPBR ± WPRT were associated with a decreased number of bone metastases in the bony pelvis compared to other treatments. In addition, there was a significant association, in the PPBR field, between EBRT and the occurrence of bone metastases, when the threshold is one bone metastasis: 19 patients (34%) in the No-EBRT group developed at least one bone metastasis, compared to 1 patient (2%) in the EBRT group (*p* < 0.01). When we consider the pelvic nodal field, we found no association between nodal EBRT and the number of pelvic bone metastases. This last point can likely be explained by the lower dose delivered to pelvic bones surrounding the pelvic nodes as compared to the pelvic bones surrounding the prostate or prostate bed. This could also be explained by a lack of power, since all patients in the EBRT group received PPBR, but only 24 patients (44%) had WPRT.

As far as we know, our study is the first to report a prophylactic effect of EBRT on the occurrence of bone metastases in the bony pelvis.

Recently, Hwang et al. showed that immediate adjuvant EBRT after RP was associated with better MFS compared to surveillance followed by early-salvage EBRT after RP (21), suggesting a better control of micro-metastatic disease, more localized immediately after first local treatment. Another recent study (14) reported the pattern of relapse after RP, and found that the bony pelvis (16%) was the first bone metastatic site, and the second site after local failure in the prostate bed (21%). In addition, when PSA is <1 ng/ml (i.e., early in the course of relapse) the bony pelvis is the most common metastatic site.

The radiation technique is decisive in dose distribution. Accordingly, 3D-EBRT is different from VMAT. The former technique is hardly used anymore, and is basically composed of four static fields, involving moderate doses to healthy tissue in radiation fields, while healthy tissues that are not in the radiation field are spared. The second technique, implemented in daily practice in our center nowadays, is more technically complex with regard to dose distribution, and relies on IMRT. In VMAT, the dose is delivered at 360 degrees using coplanar fields, allowing for better conformity of the target volume. Nevertheless, low doses are significantly diluted in healthy tissues. In our study, EBRT was delivered mainly (94%) with static fields (3D-EBRT or IMRT). We pooled 3D-EBRT and IMRT because the dose distribution is comparable, with limited low doses delivered to organs at risk and especially bony pelvis. No effect of radiation technique was observed in bone metastases distribution within bony pelvis. This could be explained by a lack of power. Nevertheless, since these two techniques have a different dose distribution, they may have a different impact on the prophylactic effect against the occurrence of bone metastases.

It has recently been shown that patients with a high number of bone metastases (>4) (22) or a high metastatic volume (23) had a lower OS. In our study, the benefit in OS was correlated with the number of bone metastases in and out of bony pelvis (≤5 or >5). The number of bone metastases can reflect the aggressiveness of cancer and its stage. In our study the number of bone metastases in bony pelvis was correlated with OS. There was no difference in OS between EBRT group and no-EBRT group.

At initial diagnosis, PSA was more important in no-EBRT group compared to EBRT group. Indeed, patients in no-EBRT group had a more advanced disease, and were less prone to have a local treatment: 70% of them received ADT alone. This is a limit related to the retrospective nature of our study, patients were not deemed comparable as in a randomized prospective study. The numbers of overall bone metastases and bone metastases out of bony pelvis were similar between no-EBRT and EBRT groups. This suggests that the initial difference of PSA between groups did not have any influence on the primary outcome, which was the number of bone metastases in EBRT field.

Although this study suffers from limitations related to its retrospective nature, it raises questions about the pathophysiology of bone metastases in previously irradiated PCa. It is noteworthy that some patients had a high PSA at initial diagnosis, both in the no-EBRT group (297.7 ng/ml) and EBRT group (290 ng/ml). These patients would have been classified as non-metastatic disease certainly because the detection threshold of imaging exams was low at the time (first patients were included in 1986). Thus, this may have induced bias, since patients who already had metastases at diagnosis, possibly in the bony pelvis, may have been treated for the primary prostate tumor with curative intent. It would be interesting to identify these high-risk patients, who may develop early occult bone metastases, by means of a risk score and/or a modern imaging such as prostate specific membrane antigen—positive emission tomograpy—(PSMA–PET) (24). In this setting, systemic treatment such as ADT, chemotherapy or metabolic radiotherapy with alpha emitter radium-223 (25) or ^177^Lu-PSMA-617 PET CT (26), could be introduced with curative intent. It might also be possible to adapt EBRT to destroy bone metastases in bony pelvis thanks to VMAT (27). Whole pelvic nodal radiation therapy is still debated for micrometastatic nodal pelvic control. Interestingly, Braunstein et al. (28) showed that ADT and WPRT were associated with improved OS although a combination of the two does not yield greater benefit. They suggested a shared mechanism for this risk reduction via the treatment of micrometastatic disease within the pelvic lymph nodes. In addition, they hypothesized that WPRT radiosensitized by neoadjuvant ADT could reduce the burden of occult micrometastases within pelvic bones, explaining the results of RTOG 94-13 (8–10). Our results need to be confirmed in prospective studies with well-balanced disease characteristics between prostate cancer patients treated or not with prostate EBRT. Given the results of this new study, it will be difficult to discard pelvic bone metastases (in field) when treating the prostate of patients with an oligometastatic prostate cancer. Our results are hypothesis generating and support enlarging pelvic fields to include pelvic bone metastases. A recent study showed that prostate EBRT to the prostate only improved significantly the OS of patients with an oligometastatic disease (23). The ongoing PEACE 1 study (NCT01957436) also aims to evaluate the benefit of prostate EBRT in the metastatic setting. For patients with an oligometastatic disease, EBRT combined with stereotactic ablative radiation therapy (SABR) is under evaluation (PEACE 6, NCT02563691). For these studies, it would be interesting to record the number of bone metastases in EBRT field to confirm or not our findings. In addition, WPRT is not realized in these studies, but extended field to the pelvis combined with SABR could improve DFS or OS. Hence, it will now be very difficult for physicians and patients to accept to discard pelvic bone oligometastases from pelvic EBRT fields in a curative intent.

## Conclusion

Prostate-bed EBRT or WPRT in PCa seems to have a protective effect against the occurrence of bone metastases in the radiation field, although the mechanism is poorly understood. These results need to be confirmed in further studies including dosimetric analyses and a pathobiological assessment of irradiated bone tissues.

## Author Contributions

MG: conceptualization, visualization, writing original draft, review, and editing. EM, MQ, LC, SL, and AC: writing review and editing. AB: formal analysis, methodology, writing review and editing. GC: conceptualization, data curation, investigation, supervision, validation, visualization, writing original draft, writing review and editing.

### Conflict of Interest Statement

The authors declare that the research was conducted in the absence of any commercial or financial relationships that could be construed as a potential conflict of interest.

## Abbreviations

ADT: Androgen deprivation therapy
CI: Confidence interval
DFS: Disease free survival
EBRT: External beam radiation therapy
HR: Hazard ratio
IMRT: Intensity modulated radiation therapy
MFS: Metastasis free survival
OR: Odds ratio
OS: Overall survival
PCa: Prostate cancer
PLND: Pelvic lymph node dissection
PPBR: Prostate or prostate bed radiotherapy
PSA: Prostate specific antigen
PSMA PET: Prostate specific membrane antigen positive emission tomography
RP: Radical prostatectomy
RTOG: Radiation therapy oncology group
SABR: Stereotactic ablative radiation therapy
SD: Standard deviation
SWOG: South west oncology group
VMAT: Volumetric modulated arc therapy
WPRT: Whole pelvic nodal radiation therapy.
